# Functional Outcome With Percutaneous Ilio-sacral Screw Fixation For Posterior Pelvic Ring Injuries In Patients Involved In Heavy Manual Laboring

**DOI:** 10.5704/MOJ.1511.003

**Published:** 2015-11

**Authors:** SM Abhishek, Prashanth, AL Azhar, GB Vijay, K Harshal

**Affiliations:** Department Of Orthopedics, Dr. Vaishampayan Memorial Government Medical College, Solapur, India

**Keywords:** Percutaneous ilio-sacral fixation, ilio-sacral screw, Majeed score, unstable posterior pelvic injuries, heavy manual laboring

## Abstract

Introduction: Unstable posterior pelvic ring injuries are best treated with operative methods due to better post-op functional score. Our patient cohort was involved in heavy manual laboring frequently required ground level work in their activities of daily living. There are very few outcome studies dealing exclusively with such patients.

Materials & Methods: Forty one patients who were treated with percutaneous sacroiliac screw fixation under fluoroscopic guidance and were followed-up for at least one year were analyzed retrospectively for functional outcome using the Majeed score.

Results: Twenty one (51.22%) and thirteen (31.70%) patients were found to be in excellent and good categories respectively and majority of the patients (thirty/73.17%) were able to return to their original occupation with or without minor adjustments.

Conclusion: Percutaneous ilio-sacral screw fixation for posterior pelvic unstable injuries is an acceptable mode of treatment in patients involved in heavy manual laboring.

## Introduction

Posterior pelvic ring (PPR) injuries are serious injuries with high rates of mortality and morbidity due to other associated injuries and due to the pelvic trauma itself. Three modes of treatment have been advocated for PPR disruptions; viz. non-operative, open reduction & fixation and percutaneous screw fixation^1^. Percutaneous screw fixation for PPR injuries has been shown to provide good biomechanical stability & functional outcome and is a good compromise between non-operative management having risk of residual disability in unstable injuries and open procedures having risk of infections due to excessive soft tissue stripping^2,1,3^. Though there are proponents of each of them, there have been very few studies focusing exclusively on the outcome of the percutaneous screw fixation in the rural populations of developing countries where unlike the Western countries, squatting, sitting cross-legged and frequent forward bending are an important component of activities of daily living and majority of the population is involved in some form of heavy manual laboring.

## Materials and Methods

A retrospective study of forty-nine consecutive patients was conducted at the authors’ institute, which is a medical college hospital and a level I trauma center in India in patients with unstable pelvic ring injuries who were treated with percutaneous ilio-sacral screw fixation in the intervening period between June 2007 to May 2014.

Ethics- The study design was reviewed and approved by the Departmental & Institutional Ethics and Review Board. Informed consent was taken in all patients for use of their clinical data/imaging and for subsequent follow-up examinations and radiology.

Study Design- It was a retrospective analysis in which all the patients meeting the inclusion criteria and consenting to be included in the study were considered.

Inclusion Criteria-

1. Traumatic unstable posterior pelvic injury based on Young-Burgess classification system (lateral compression or LC type II and III, antero-posterior compression or APC type II and III, vertical shear or VS and combined injury patterns which were unstable)
2. Skeletally mature
3. Minimum one year of follow-up at the time of study after the surgery
4. Occupation involving heavy manual laboring and ground-level activities

Exclusion criteria-

1. Injuries which needed open reduction, either primarily or in the follow-up period for revision
2. Skeletally immature
3. Associated acetabular injuries
4. Concomitant long bone fractures of lower limb
5. Spinal cord injuries

Forty nine patients (Table I) were fitting in the inclusion criteria at the start of the study out of which four were lost to follow-up, two died due to unrelated causes midway during the study and two necessitated open reduction due to increased displacement in the follow-up period (cases requiring revised percutaneous screw fixation were included in the study). So forty-one patients were included in the final analysis.

**Table I:** Demographic data and classification

	Variables	Absolute number (41)	Percentage
Gender			
	• Male	26	63.41%
	• Female	15	36.59%
Injury side			
	• Right	22	53.66%
	• Left	19	46.34%
Mechanism of Injury			
	• Fall	7	17.07%
	• Road traffic accident	34	82.93%
Employment status			
	• Working	41	100%
	• Not working	0	0%
Young-Burgess types			
	• LC*-II	6	14.63%
	• LC-III	5	12.20%
	• APC#-II	7	17.07%
	• APC-III	6	14.63%
	• VS§	15	36.59%
	• Combined Patterns	2	4.88%

* Lateral compression

# Anterior‐posterior compression

§ Vertical shear

After routine pre-anaesthetic check-up, the patient was taken up for ilio-sacral screw fixation using 6.5 mm partially threaded cannulated cancellous screws with washers. One or two screws were used under fluoroscopic guidance depending on the bony anatomy of the patient and the subjective feel of stability by the surgeon^4^. Anterior fixation, if needed, was done in supine position was done later. No intraoperative electro diagnostic studies for potential nerve damage were done. For PPJ fixation the guide wire for the screw was placed below the iliac-cortical density at the posterior cortex of the sacral body in the lateral fluoroscopic view. Then the outlet view was taken to direct the guide wire in a cephalad direction above the S1 foramen towards the superior endplate. Finally the inlet view was taken to make sure that the trajectory is directed towards sacral promontory. The guidewire was advanced till it just crossed the midline. A screw was then inserted over it using a stab incision^5,6^. The reader is referred to the pre-operative and intra-operative fluoroscopic images in the adjoining figures for better clarification (Figure 1A & 1B). All patients were discharged after two days of surgery unless there were other indications to not do so. Hip and knee range of motion exercises were started a day after surgery. The patient was kept non-weight bearing for six weeks followed by partial weight bearing for another six weeks after which the patient was allowed to walk normally without any assistance. Squatting, sitting cross-legged or forward bending (prayer/namaz position) were allowed after surgery as tolerated by the patient, provided there was no weight bearing on the affected side. Patients were allowed to resume sexual activity after six-eight weeks depending on the status of his pain. Resumption of earlier job/weight lifting/non-contact sport was allowed after a minimum of twelve weeks after surgery. Serial radiographs were obtained immediately after the surgery, after six weeks, after three months and after one year in all cases. Majeed functional score and Majeed pelvis grade were calculated at end of one year of follow-up. Where needed appropriate statistical analysis was used.

**Fig. 1:**
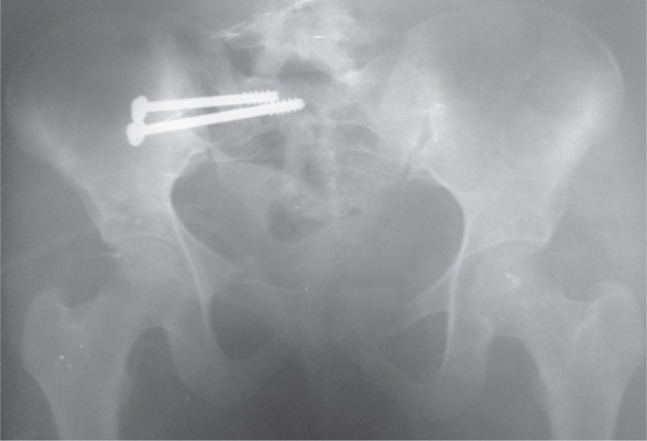
Unilateral SI joint fixation with two percutaneous cancellous screws.

**Fig. 1a:**
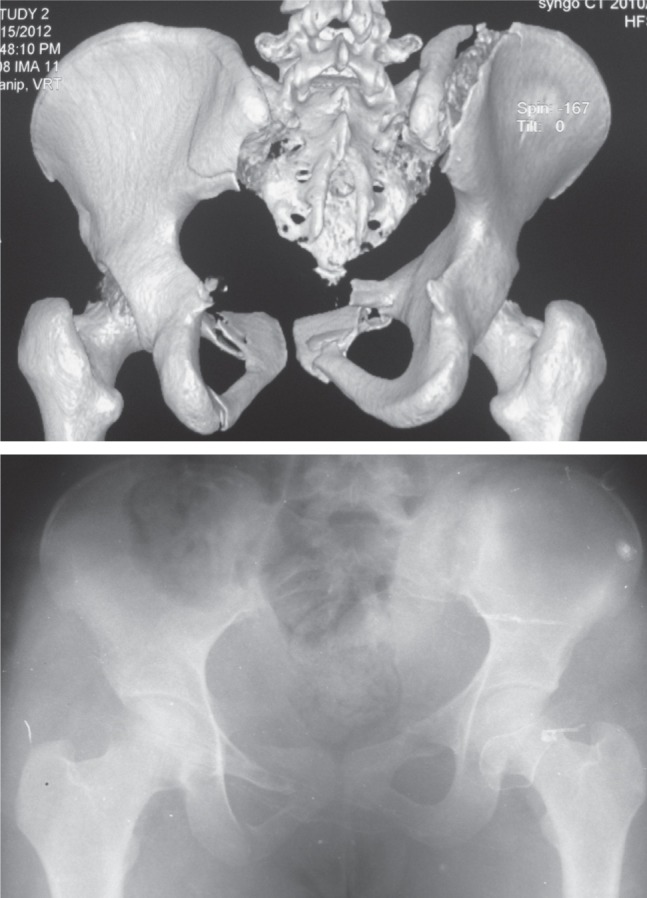
Pre-operative 3D CT scan 1B : Plain Antero-posterior radiograph respectively.

**Fig. 1c:**
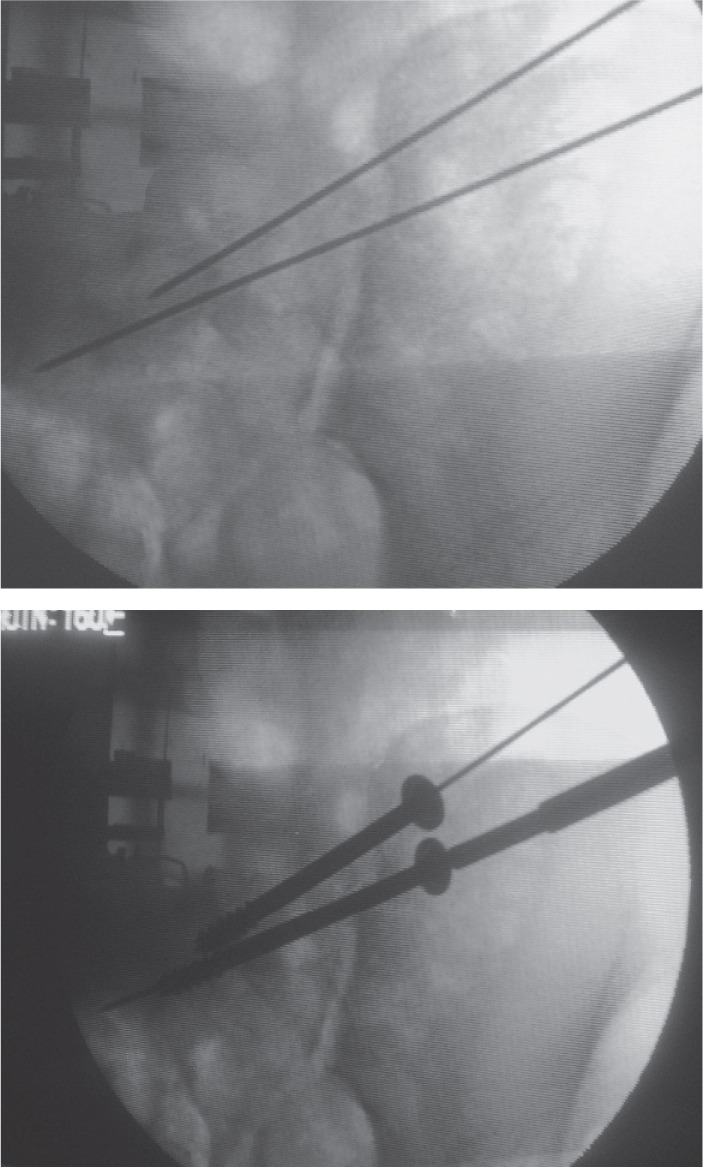
Intra-operative placement of guide-wires and screws (Attempted Antero-posterior and Outlet, views respectively).

## Results

The mean Majeed functional score for individual components is tabulated as follows: (Table II). Thirty four (82.92%) patients were in the excellent or good category of the Majeed pelvic grade (Table III). Thirty (73.17%) patients were able to go back to their original occupation with minor adjustments at the time of follow-up at end of one year. Two (4.88%) patients had superficial infection at the site of screw insertion, which resolved by local debridement and antibiotics. Two (4.88%) patients were operated with revision percutaneous screw at six weeks due to loss of reduction and resultant vertical pelvic displacement as measured by Henderson’s criteria of more than five mm^2^. One (2.43%) patient had tingling in the posterior part of thigh, which resolved on its own in eight weeks. There were no cases of motor nerve damage, vascular/ureteral injury, sexual dysfunction, or deaths related to the surgical procedure. (Figure 2)

**Table II:** Majeed functional score

Score (Maximum value)	Mean value
Standing (36)	29.76
Pain (30)	23.78
Work (20)*	16.53
Sitting (10)	8.19
Sexual intercourse (4)	2.83
Total score	81.09

* All the patients in our cohort were working, either full-time or part-time.

**Table III:** Majeed pelvic grade

**Grade**	**Total Cases (n/percentage)**
Excellent	21 (51.22%)
Good	13 (31.70%)
Fair	4 (9.76%)
Poor	3 (7.31%)

## Discussion

The unique advantage of percutaneous ilio-sacral screw fixation is simplicity of the procedure with minimal blood loss, less indoor time and minimal cost^2^. All patients were routinely discharged after forty eight hours which firstly reduces the patient load at the institutes like ours which has very high patient load and secondly reduces the financial burden on the patients and their families, majority of whom are from lower socioeconomic background. Compared to conservative and open procedures, we feel the patients had better pain relief probably due to early pelvic stabilization, minimal instrumentation and less soft tissue trauma^1,7^.

Conservative management is a poor choice especially in our case cohort because majority of them were daily-wage workers & laborers. The time for recovery for conservative method has been shown to be greater than operative methods in many studies and also the functional outcome in the former is poorer as compared to the later^1,2^.

The open reduction though might give an anatomical reduction and maybe a greater strength to fixation, incase plates were used; the technique is not without complications. There is blood loss, an increased risk of infection and increased cost due to increased operative time^3,7,8^.

Though percutaneous technique is relatively blind and logically it may be felt that there might be greater instance of iatrogenic injury, no such complications occurred in our series. This may be attributed to strict adherence to the intraoperative fluoroscopic bony landmarks for screw placements^5,6,9^.

Majeed functional score and pelvic grade score is the most widely used scoring system for the SIJ injuries^10^. Majority of our patients (82.92%) were in excellent or good category, which is in sync with other studies in these types of injuries^1,2,11–13^. Again this can be attributed to strict adherence to the inclusion criteria. We had a very low threshold for abandoning the procedure and opting for open reduction incase of non-satisfactory joint reduction.

Though Majeed functional score covers majority of the activities involving usage of SIJ, the score falls short in assessing certain activities which are considered essential in many developing and under-developed countries- squatting, sitting cross legged and forward bending (prayer/namaz position). These are the very activities, which were of importance in our cohort of patients, majority of whom were manual laborers and belonging to low socio-economic strata (Figure 3A,3B,3C). Though these activities are not reflected directly in the Majeed scoring system, the final outcome as mentioned earlier is concurrent with other studies.

**Fig. 3a:**
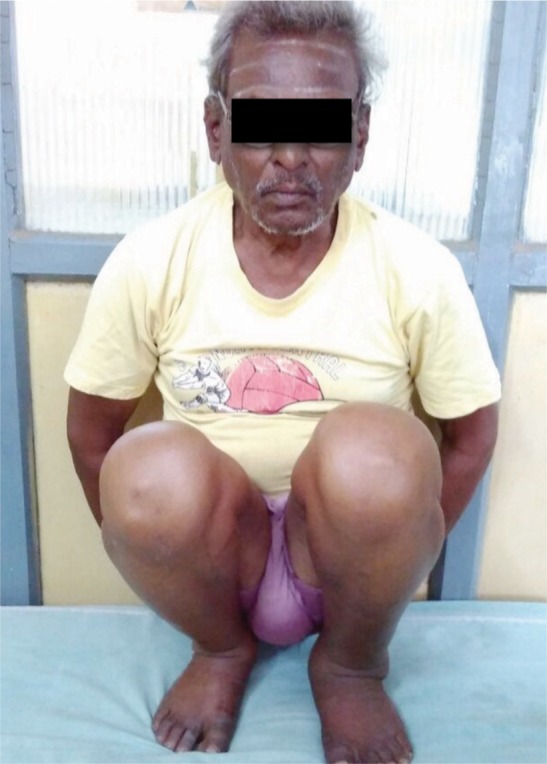
Squatting.

**Fig. 3b:**
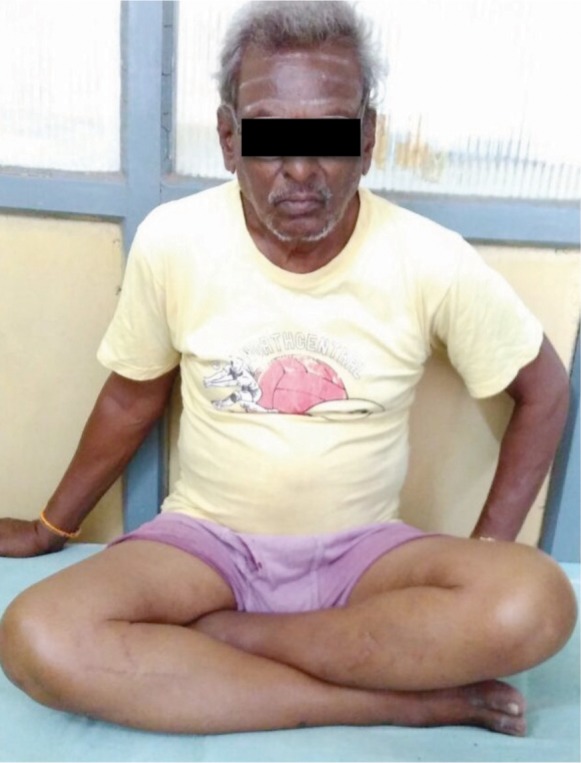
Sitting cross-legged.

**Fig. 3c:**
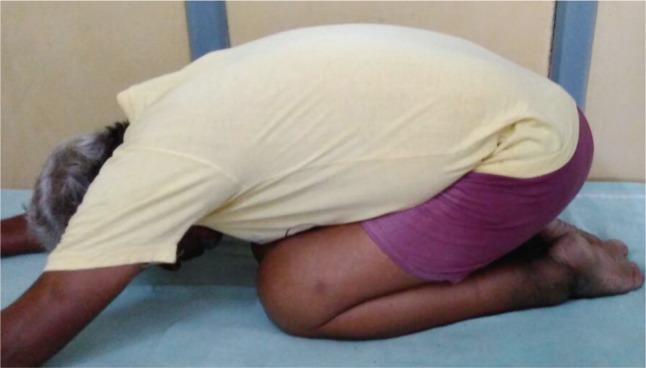
Forward bending (Prayer/ Namaz) position.

The major limitation of the study is its small sample size (because of the rarity of the injury pattern). The validation of the Majeed score in the patient cohort as ours will need larger study population ideally involving multiple centers. Also there was no control group or any comparison with other modality of treatment to test the utility of the score and the outcome for percutaneous screw fixation in unstable SIJ injuries in them.

## Conclusion

To conclude, it can be said that percutaneous screw fixation of unstable PPR injuries in patients requiring squatting, sitting cross-legged and forward bending (prayer/namaz position) and involved in heavy manual laboring can give satisfactory result.
